# Carotenoid composition and antioxidant potential of *Eucheuma denticulatum*, *Sargassum polycystum* and *Caulerpa lentillifera*

**DOI:** 10.1016/j.heliyon.2020.e04654

**Published:** 2020-08-12

**Authors:** V. Balasubramaniam, L. June Chelyn, S. Vimala, M.N. Mohd Fairulnizal, I.A. Brownlee, I. Amin

**Affiliations:** a Nutrition, Metabolism & Cardiovascular Research Centre, Institute for Medical Research, Ministry of Health Malaysia, Level 3, Block C7, No.1, Jalan Setia Murni U13/52, Setia Alam, 40170, Shah Alam, Selangor, Malaysia; b Herbal Medicine Research Centre, Institute for Medical Research, Ministry of Health Malaysia, Level 3, Block C7, No.1, Jalan Setia Murni U13/52, Setia Alam, 40170, Shah Alam, Selangor, Malaysia; c Forest Research Institute Malaysia, 52109, Kepong, Selangor Darul Ehsan, Malaysia; d Faculty of Health and Life Sciences, Northumbria University, Newcastle-upon-Tyne NE1 8ST, UK; e Department of Nutrition and Dietetics, Faculty of Medicine and Health Sciences, Universiti Putra Malaysia, 43400, Serdang, Selangor, Malaysia

**Keywords:** Food science, Food technology, Food analysis, Nutrition, *Caulerpa lentillifera*, *Sargassum polycystum*, Orbitrap, LC-MS/MS, Carotenoid, *Eucheuma denticulatum*, Antioxidant

## Abstract

Three species of Malaysian edible seaweed (*Eucheuma denticulatum, Sargassum polycystum and Caulerpa lentillifera*) were analyzed for their carotenoid composition using a combination of high-performance thin layer chromatography (HPTLC) and ultra-high-performance liquid chromatography-electrospray ionization-tandem mass spectrometry (UHPLC-ESI-MS/MS), while the antioxidant capacities were determined by 2,2-diphenyl-1-picrylhydrazyl (DPPH) and oxygen radical absorbance capacity (ORAC) assays. The HPTLC analysis exhibited a distinct carotenoid pattern among the three seaweed groups. The UHPLC-ESI-MS/MS analysis showed fucoxanthin as the major carotenoid present in *S. polycystum* while lutein and zeaxanthin in *E. denticulatum*. For *C. lentillifera*, β-carotene and canthaxanthin were the major carotenoids. Some of the carotenoids, such as rubixanthin, dinoxanthin, diatoxanthin and antheraxanthin, were also tentatively detected in *E. denticulatum* and *S. polycystum*. For antioxidant activity, *S. polycystum* (20 %) and *E. denticulatum* (1128 μmol TE/g) showed the highest activity in the DPPH and ORAC assays, respectively. The findings suggest the three edible varieties of seaweeds may provide a good dietary source with a potential to reduce antioxidative stress.

## 1 Introduction

Seaweed remains a popular food ingredient in many parts of Asia and is an important source of high-value hydrocolloids, such as agar, alginates and carrageenan [1, 2]. In addition, seaweeds are increasingly recognized for their health promoting benefits, leading to a growing interest and number of utilization in the nutraceutical, pharmaceutical and cosmetic industries [3]. Previous studies have further shown that seaweed-derived extracts and compounds possess many interesting bioactivities, such as anti-inflammatory, anti-diabetic, anti-obesity and anti-cancer properties [4, 5, 6]. The observed activities could partly be contributed by the range of antioxidant compounds found in seaweeds [7, 8, 9, 10].

Seaweeds are exposed to many environmental factors that favor the generation of free radicals and strong oxidizing agents [11, 12, 13]. As a result of these harsh conditions, seaweeds are highly resistant to oxidative damage, possibly contributed by the antioxidant compounds within their cells [14].

Carotenoids are one of the essential antioxidant compounds in seaweeds [15]. Carotenoids, which have a characteristic linear C40 chain, contain up to 11 conjugated bonds (allenic bonds) that may participate in antioxidant activities via the transfer of the excess energy of singlet oxygen (O•) in the long central allenic chain [16]. Moreover, the allenic bonds and other functional groups at the terminal rings of the structure, as seen in astaxanthin and fucoxanthin, may also react with free radicals and thus further contribute to its antioxidant potential [9]. Although profiling of carotenoids has been used for the taxonomic classification of seaweed, data on their concentrations in different phyla remain scarce.

Traditionally, the separation and characterization of carotenes (nonpolar carotenoids) and xanthophylls (polar carotenoids) have been achieved using thin-layer chromatography (TLC) and high-performance liquid chromatography coupled to a UV or photodiode array detector (HPLC-UV/DAD) [15]. However, the complexity of the extract matrices, as well as close structural similarities between the carotenoids, necessitates new methods that are more precise and sensitive to distinguish closely related isomers and accurately quantify them [15, 17]. The development of high-resolution and accurate mass spectrometry (MS) has allowed complex mixtures of carotenoids to be distinguished according to their accurate masses and fragmentation patterns [18]. Various MS ionization methods have also been investigated in the analysis of carotenoids, such as atmospheric pressure chemical ionization (APCI) and the electrospray ionization (ESI) method [19, 20]. Moreover, substantial improvements have been made in the development of ultra-high-performance liquid chromatography (UHPLC), which allows better sensitivity and a shorter run time for carotenoid analysis [18, 21].

To date, information regarding the bioactive components present in the Malaysian local seaweeds remain insufficient. The current work is a continuation of our previous research [4, 5]. These studies reported that the ethanol extracts of *E. denticulatum* manifest anti-diabetic, anti-obesity and anti-inflammatory properties. The health benefits shown in the ethanol extracts may be due to the presence of polyphenols or other antioxidant components, such as carotenoids. The evaluation of polyphenols content has been reported previously on the selected seaweeds [22, 23]. Therefore, in the present study, we investigate the carotenoid composition of *E. denticulatum* (red seaweed), *S. polycystum* (brown seaweed) and *C. lentillifera* (green seaweed) and their antioxidant capacity. Brown and green seaweed were included for comparison with the red seaweed for this study. A rapid, HPTLC system was used for the quick visualization and identification of carotenoids in the different seaweeds, while UHPLC coupled to an ESI-MS detector (UHPLC-ESI-MS) was used to further validate and quantify the identified carotenoids. The antioxidant activities were assessed by the 2-diphenyl-2-picrylhydrazil (DPPH) radical scavenging assay and oxygen radical absorbance capacity (ORAC) assay.

## 2 Materials and methods

### 2.1 Chemicals

HPLC-grade ethanol (EtOH) and dichloromethane were purchased from Merck (Merck Co., Darmstadt, Germany). The LC-MS-grade solvents acetonitrile (ACN), methanol (MeOH) and formic acid (FA) were purchased from Fisher Chemical (New Jersey, USA). Chemical standards of lutein, β-carotene, zeaxanthin, and lycopene were purchased from Sigma (St Louis, MO, USA); those of β-cryptoxanthin, all-trans-canthaxanthin, all-trans-fucoxanthin, all-trans-astaxanthin, violaxanthin and neoxanthin were obtained from ChromaDex (Santa Ana, CA). All other reagents and solvents were of high analytical grade unless otherwise stated. Water was obtained from arium® pro Ultrapure Water Systems (Sartorius, Goettingen, Germany). All solvents were degassed before use.

### 2.2 Seaweed collection and processing

The seaweeds (*E. denticulatum, S. polycystum* and *C. lentillifera*) were collected from the coast of Sabah with the assistance of the Aquaculture Division, Department of Fisheries, Malaysia. Samples were processed immediately upon arrival at the Institute for Medical Research, Malaysia, as previously discussed [4, 5]. Briefly, the collected seaweed samples were cleaned thoroughly to remove epiphytes, salt and sand using tap water and finally rinsed with deionized water three times before being cut into small pieces. The samples were placed in glass jars and sealed with parafilm. Then, the seaweeds were frozen at -80 °C prior to freeze drying at -85 °C (Beta 2–8 LDplus freeze dryer, Martin Christ, GMBH, Germany) and ground to a fine powder (40 mesh size) using a Waring blender. Samples were stored in airtight containers at -80 °C until further analysis.

### 2.3 Sample preparation

Freeze-dried seaweed powder was mixed in a ratio of 1:20 w/v with the extraction solvent (100 % ethanol; EMSURE® ACS) by stirring at room temperature for 24 h. The crude extracts were obtained by ﬁltration using a Whatman filter paper (No. 1), and the filtrate was concentrated using a rotary evaporator (Buchi Rotavapor R-200, Switzerland) under reduced pressure at 45 °C. The concentrated extracts were subsequently aliquoted into a 5 ml Bijou bottle. Next, the extracts were dried overnight (45 °C) by vacuum centrifugation using MiVac (Genevac, UK) to yield a dark-green extract for *E. denticulatum* and *C. lentillifera*, while for *S. polycystum*, a dark-brown extract was obtained. The extracts were maintained at -20 °C until further use. Each so-obtained dried pellet (seaweed extract) was reconstituted with relevant solvents according to analysis. For the LC-MS analysis, the seaweed extract was diluted with a 50:50 DCM/MeOH solution and then filtered through a 0.22 μm nylon ﬁlter into a LC vial to remove any impurities before loading to the UHPLC. A concentration of 1 mg/ml was injected in UHPLC.

### 2.4 Preparation of stock standard solutions

Stock standard solutions (10 mg/ml) were prepared individually by dissolving the analytes in a 50:50 DCM/MeOH solution. The solutions were stored in the dark at -20 °C. The standard stock solution was used for spiking, as different spiking levels are required for validating the method. Only primary grade standards (~99.5 % purity) were used for the LC-MS/MS analysis.

### 2.5 Identification of carotenoids

#### 2.5.1 Carotenoid analysis by HPTLC

HPTLC analysis was performed using a HPTLC plate precoated with silica gel 60 F_254_ (Merck, Darmstadt, Germany). Briefly, ethanolic extracts of each seaweed (*E. denticulatum*, *C. lentillifera* and *S. polycystum*) (10 mg) and standards (1 mg) were dissolved in a 50:50 DCM/MeOH solution by sonication to make 10 mg/ml and 1 mg/ml stock solutions, respectively. The crude extract solution (10 μl) and standard solutions (2 μl) were applied to the HPTLC plate as 8 mm-wide bands using a Camag LINOMAT V applicator (Muttenz, Switzerland). A HPTLC developing chamber was presaturated with the mobile phase consisting of toluene: acetone (70:30, v/v) for 20 min. Then, the HPTLC plate was inserted into the chamber and developed until the developing distance reached 70 mm. Finally, the plate was removed from the chamber, air-dried and visualized under white light without any further derivatization.

#### 2.5.2 Carotenoid analysis by UHPLC-ESI-MS/MS

Carotenoids were identified according to a modified method described previously [24] using a UHPLC-ESI/HRMS/MS^n^ system that consisted of a Dionex Ultimate 3000 UHPLC system (Thermo Scientific, CA, USA) and a Q Exactive Hybrid Quadrupole-Orbitrap Mass Spectrometer (Thermo Scientific, CA, USA). A MS-compatible C18 reversed-phase column (50 mm × 2.1 mm i.d., 1.7 μm particle size), Acquity UPLC BEH C18 (Waters, MA, USA), was used in this study. The autosampler was set at 4 °C, and sample volumes of 3 μl were loaded onto the column. The column was run at a temperature of 35 °C. The gradient program was composed of two mobile phases, water with 0.1 % formic acid (A) and ACN with 0.1 % formic acid (B). The flow rate was 0.3 ml/min, and the gradient program was as follows: 0–2 min, 5 % B; 2–20 min, from 5 % B to 99 % B, 20–25 min, 99 % B; 25–30 min 5 % B. The MS analysis was carried out in positive and negative ion electrospray ionization (ESI) modes. The mass spectrometer was used with a resolving power setting of 140,000 FWHM and a scan range of *m/z* 100–1000, the capillary temperature was set at 320 °C, and the sheath gas and auxiliary gas flow rates were 35 and 12 arbitrary units, respectively. The spray voltage was at 3.7 kV. The S-tube lens was set at 55 V for both the positive and the negative ionization modes. The MS/MS spectra were acquired using a collision energy of 35 V. Data were generated using X-calibur 2.1.0 (Thermo Scientific, CA, USA). A column guard was used to avert damages and blockage in the column due to the complex nature of seaweed. In addition, washing steps were taken between long sample runs to prevent the column from becoming dirty.

#### 2.5.3 Peak identification

Compound identification was based on accurate mass determination at a resolution of 140,000 and additional higher-energy collision dissociation (HCD) fragments with a mass deviation below 5 ppm. Most peaks were identified using standards by comparing the chromatographic retention times. A tentative identiﬁcation of compound based on a comparison with data in the literature was applied when no standards were available.

#### 2.5.4 UHPLC-MS/MS method validation

A quantitation method using Thermo Scientiﬁc Q-Exactive Xcalibur 2.2 software, which combines chromatography with high-resolution mass spectrometry, was developed for the determination of astaxanthin, β-cryptoxanthin, canthaxanthin, lutein, fucoxanthin and zeaxanthin. The validation procedure was performed through determination of the linearity, limit of detection (LOD), limit of quantiﬁcation (LOQ) and recovery. To ascertain the validity of the retention times, standards were run frequently. Quantitative calibration was carried out using a ﬁve-point calibration.

### 2.6 Antioxidant capacity

#### 2.6.1 2-Diphenyl-2-picrylhydrazil (DPPH) radical scavenging assay

The scavenging capacity of the DPPH radical was estimated according to the method of Blois [25] with a modification using a microplate system. A sample (50 μl of 1.0 mg/ml) was added to 50 μl of DPPH (1 mM in ethanolic solution) and 150 μl of ethanol (absolute) in a 96-well microtiter plate in triplicate. The plate was shaken at 500 rpm for 15s and followed by incubation at room temperature for 30 min. Finally, the absorbance was measured spectrophotometrically at 520 nm.

#### 2.6.2 Oxygen radical absorbance capacity (ORAC)

The ORAC assay was performed as described previously [26] with some modifications. AAPH (2,2′-azobis (2-amidino-propane) dihydrochloride) (0.65 g) was dissolved in 10 ml of 75 mM phosphate buffer (pH 7.4) to a final concentration of 240 mM (made fresh daily). A fluorescein stock solution (1 mM) was made in 75 mM phosphate buffer (pH 7.4) and stored at 5 °C, wrapped in foil. Immediately prior to use, the stock solution was diluted 1:100,000 with 75 mM phosphate buffer (pH 7.4) (made fresh daily). To all experimental wells, 150 μl of working sodium fluorescein solution was added. The blank wells were added with 25 μl of Trolox 6-hydroxy-2, 5, 7, 8-tetramethylchroman-2-carboxylic acid (Trolox) dilution. The sample wells were added with 25 μl of samples. Following that, the plate was equilibrated by incubating for 10 min at 37 °C. A BMG Omega Fluostar™ fluorescent spectrophotometer with an injector was used with an excitation filter of 485 nm and emission filter of 528 nm. Reactions were initiated by the addition of 25 μl of AAPH solution (240 mM) using the microplate reader's injector for a final reaction volume of 200 μl. The plate was shaken at maximum intensity for 50 s. The fluorescence was then monitored kinetically with data taken every minute. The fluorescence of each well was measured every 60 s. ORAC values were calculated using MARS Data Analysis Reduction Software.

### 2.7 Statistical analysis

Data were analyzed using GraphPad Prism version 5.0 for Windows (San Diego, California, USA). Seaweed extraction and analysis were performed in triplicate for each sample. Quantitative results were expressed as mean ± SD. A comparison of means was performed by one-way ANOVA, and a significant difference was accepted at a probability of *p* < 0.05 determined by Bonferroni's test.

## 3 Results and discussion

### 3.1 Carotenoid composition in *E. denticulatum*, *S. polycystum* and *C. lentillifera* by HPTLC analysis

**Figure 1:**
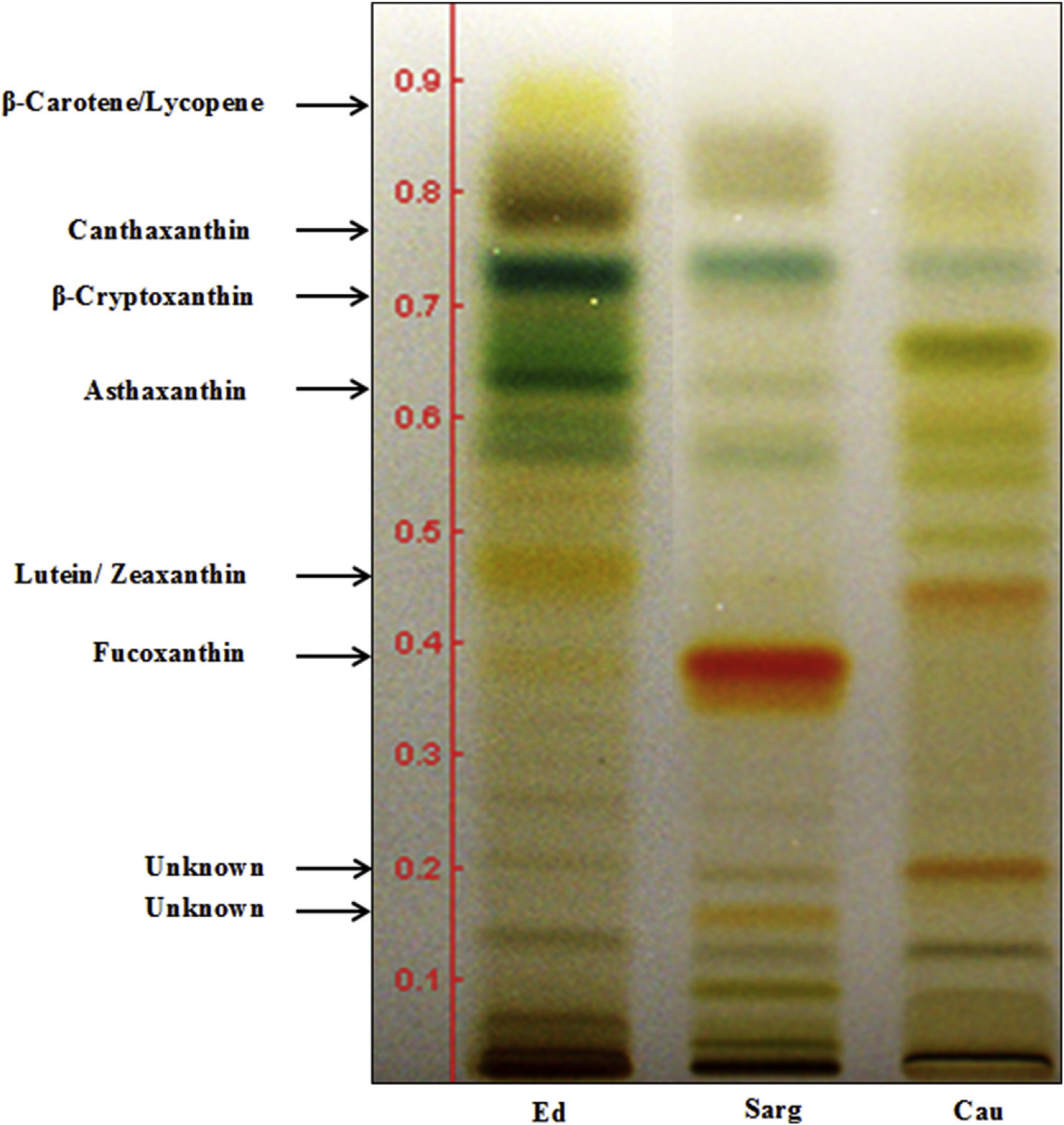
HPTLC analysis of carotenoids in ethanolic extracts of red seaweed, *E. denticulatum* (Ed), brown seaweed, *S. polycystum* (Sarg) and green seaweed, *C. lentillifera* (Cau) developed on a silica gel HPTLC plate with toluene: acetone (70:30,v/v) as the mobile phase (Full picture is provided in supplementary 1).

Carotenoids are usually categorized into two groups: xanthophylls, which contain oxygen in their molecular structure, and carotenes, which are hydrocarbons. Both groups absorb light at different wavelengths during photosynthesis, and hence, xanthophylls appear more yellow, while carotenes are orange. In this study, HPTLC was used to analyze the color profiles and distribution pattern of carotenes and xanthophylls, as well as to tentatively identify the carotenoids present in the selected seaweed. According to Giri et al. [27], HPTLC-based solute separation can be useful in the development of validation and standardization processes, as well as isolation of pure compounds and facilitate the determination of classes of compounds in plants. Hence, seaweed fingerprinting is helpful in identifying a seaweed. A comparison of the carotenoid contents in the three species of seaweed by HPTLC is shown in Figure 1. The HPTLC patterns of the three seaweeds suggest the presence of distinct phytochemicals, where the major components detected were carotenoids along with chlorophylls and pheophythins. The HPTLC results clearly demonstrate that the seaweeds manifest a class-specific composition of xanthophylls. *E. denticulatum* contains mainly zeaxanthin or/and lutein (R_f_ = 0.44) and β-carotene (R_f_ = 0.88), present as light-yellow bands. Another tentatively detected xanthophyll, β-cryptoxanthin, is an intermediate in the biosynthesis pathways of lutein or zeaxanthin. A similar finding was reported by Marquardt and Hanelt [28]. Additionally, fucoxanthin, which is commonly found in brown seaweed, was detected in this red seaweed as a light-yellow band, indicating a smaller concentration compared to that of fucoxanthin in *S. polycystum* (brown seaweed), which appeared as a bright orange band. According to previous findings [29, 30], red seaweed (Rhodophyta) does not present a unique carotenoid profile; nonetheless, the ubiquitous carotenoids β-carotene and/or α-carotene and their dihydroxylated derivatives, zeaxanthin and lutein, were detected, which agrees with our finding.

Fucoxanthin at R_f_ = 0.39 was detected as the major xanthophyll in *S. polycystum*, which was consistent with scores of previous studies reporting the abundant presence of fucoxanthin in brown seaweeds [28, 29]. Fucoxanthin was also noted as an intermediate of the lutein and zeaxanthin biosynthesis pathways [28].

The HPTLC analysis of *C. lentillifera* (green seaweed) showed it to be similar to higher plants in that zeaxanthin or/and lutein (R_f_ = 0.44) were the dominant xanthophyll(s) present [31]. Other xanthophyll, such as canthaxanthin, was found in trace amounts in *C. lentillifera*, and this finding is in agreement with the results of Goodwin [32]. In contrast, β-carotene was not detected in the HPTLC analysis of *C. lentillifera*, as predicted based on previous findings [15]. The green pigments present in the analysis of *C. lentillifera* were probably chlorophylls and pheophythins involved in photosynthesis, which are generally found in green seaweeds as well as green terrestrial plants [33]. Hence, this finding suggests that different species of seaweed contain different kinds of carotenoids based on the phylum, which has been affirmed by previous studies; however, certain carotenoids were absent in contradiction to other findings [15, 33], which can be explained in terms of the different methods and species used.

### 3.2 Identification and quantitation of carotenoids

MS (ESI) was applied to confirm the identities of carotenoids from all three seaweed ethanolic extracts that were tentatively identified by HPTLC fingerprinting. These carotenoids were eluted under gradient conditions, and their identities were confirmed by their retention times, the accurate mass (*m/z* value) of their generated ions and the MS/MS patterns of their respective reference standards. The corresponding ^13^C isotopic ion was also monitored as additional information for determining a compound's identity. Studies have demonstrated that isotope abundances provide increased confidence of identification [34, 35]. Nevertheless, the instability of carotenoids due to factors such as heat, light and oxygen may create challenges in the identiﬁcation and quantitation of these compounds. Therefore, abovementioned factors should be taken account during sample preparation and analytical steps to ensure accurate analytical results.

**Figure 2:**
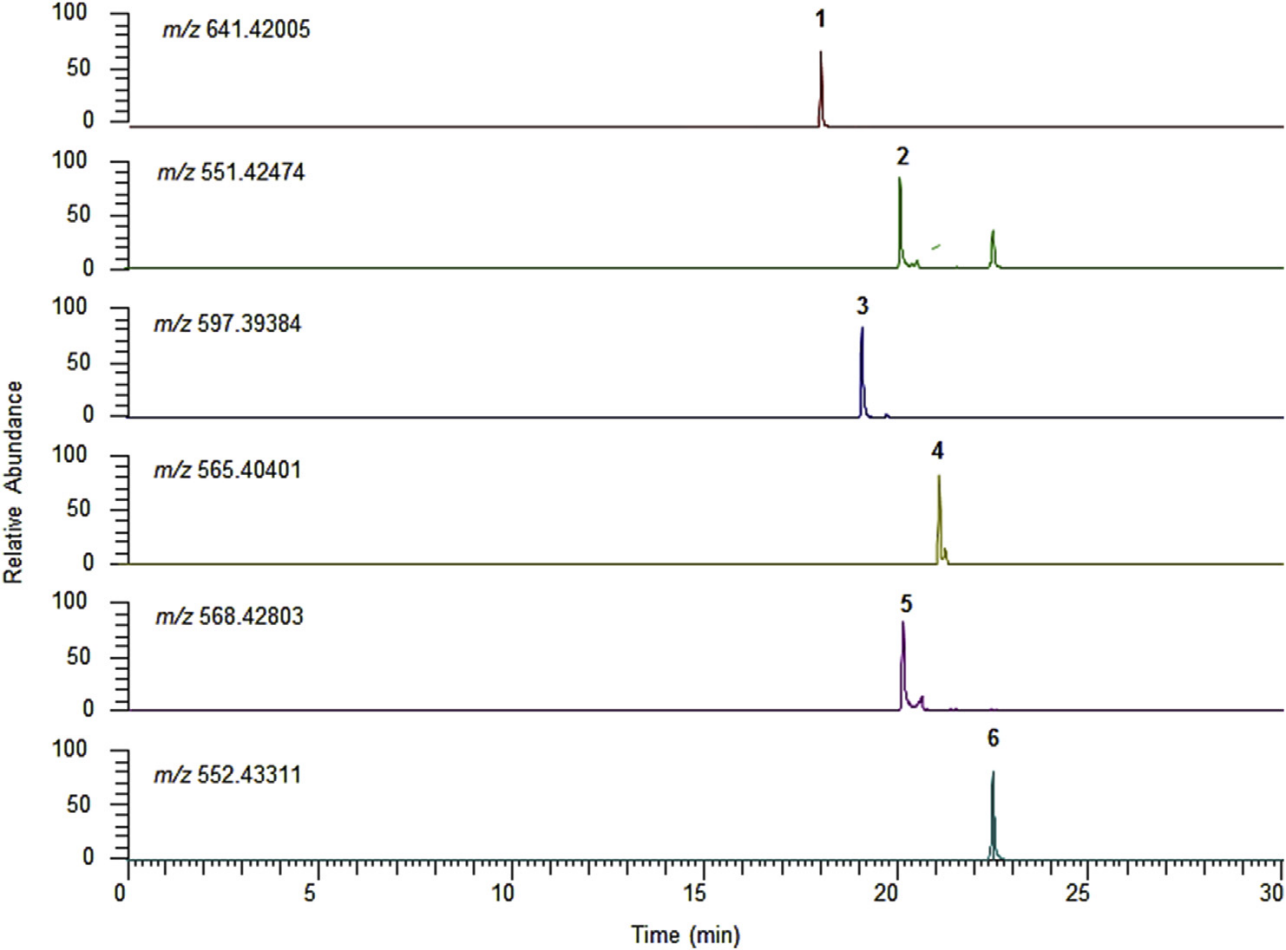
Chromatograms for selected carotenoids by gradient reversed-phase UHPLC full scan Orbitrap-MS. Peak 1, fucoxanthin; Peak 2, lutein; Peak 3, astaxanthin; Peak 4, canthaxanthin; Peak 5, zeaxanthin and Peak 6, β-cryptoxanthin. Further details of the peaks are described in Table 1.

Individual carotenoids were identified by ESI in positive ionization mode. In addition, the presence of generated molecular ions, particularly protonated [M + H]^+^, dehydrated [M + H–H_2_O]^+^ and the radical molecular ion [M]^+•^, was studied (Table 1). From the MS analysis (Figure 2), it was observed that lutein (Peak 1) forms predominantly the dehydrated molecule (i.e., [M + H–H_2_O]^+^), and this finding agrees with previously reported data [36], while for fucoxanthin (Peak 2), the dehydrated molecule *m/z* 641 [M + H–H_2_O]^+^ was selected to facilitate the comparison with the standard, although it is noteworthy that the protonated ion (*m/z* 659) was detected abundantly in the sample. For astaxanthin and canthaxanthin (Peaks 3 and 4), the protonated molecule [M + H]^+^ was noted as the most abundant species, while zeaxanthin (Peak 5) and β-cryptoxanthin (Peak 6) were characterized by the molecular ion M^+^ due to loss of one electron and formation of a stable radical system. Notably, in this study, the MS system applied with an ESI positive mode ionized xanthophylls (astaxanthin, β-cryptoxanthin, canthaxanthin, fucoxanthin and lutein) well but did not ionize hydrocarbon carotenes (such as lycopene and β-carotene). Hence, UHPLC- diode array detector was utilized to detect and quantify the β-carotene present in all three seaweed species tested.

**Table 1:** Measured theoretical and accurate masses of the targeted carotenoids in *E. denticulatum*, *S. polycystum* and *C. lentillifera* by Orbitrap-MS (identity was confirmed by standard compound).

Peak	Retention time (min)	Selected ion	*m/z*^a^ experimental	*m/z*^a^ observed	Error (ppm)	Compounds
1	17.99	[M + H–H_2_O]^+^	641.42005	641.41809	-3.05	Fucoxanthin
2	20.11	[M + H–H_2_O]^+^	551.42474	551.4237	-3.21	Lutein
3	19.10	[M + H]^+^	597.39384	597.39185	-2.52	Astaxanthin
4	21.17	[M + H]^+^	565.40401	565.40167	-3.05	Canthaxanthin
5	20.14	[M]^+•^	568.42803	568.42560	-3.31	Zeaxanthin
6	22.50	[M]^+•^	552.43311	552.43036	-3.99	β-Cryptoxanthin

a *m/z* (relative abundance).

The carotenoid concentrations of the three species of seaweeds are shown in Table 2. The detectable carotenoids for *E. denticulatum* (red) consisted of lutein, zeaxanthin, β-cryptoxanthin, and β-carotene with a few unsubstantiated carotenoids. Lutein was present as the major xanthophyll for this class of seaweed with a concentration of 87.7 mg/100 g DW. This result agrees with the findings of Marquardt and Hanelt [28], who describe lutein, in general, as the predominant carotenoid in most red seaweeds. β-Cryptoxanthin (3.6 mg/100 g) was the least abundant detected carotenoid in *E. denticulatum* at *m/z* 552. This adduct species was selected for quantitating β-cryptoxanthin due to its better peak resolution and higher abundancy compared to those of the peak at *m/z* 535.

**Table 2:** Carotenoid contents in *E. denticulatum* (red seaweed)*, S. polycystum* (brown seaweed) and *C. lentillifera* (green seaweed).

	Carotenoid concentration (mg/100 g of DW extract)						
	Lutein	Zeaxanthin	Fucoxanthin	β-Cryptoxanthin	Canthaxanthin	Astaxanthin	β-Carotene
*E. denticulatum*	87.7 ± 0.1^a^	21.3 ± 0.1^a^	4.0 ± 0.0^a^	3.6 ± 0.0^a^	<0.001	3.0 ± 0.0^a^	4.7 ± 0.1^a^
*S. polycystum*	11.7 ± 0.0^b^	13.9 ± 0.0^b^	2740.0 ± 0.5^b^	6.1 ± 0.0^ab^	21.7 ± 0.0^a^	25.5 ± 0.0^b^	<0.04
*C. lentillifera*	<0.02	3.6 ± 0.0^c^	<0.001	1.3 ± 0.0^ac^	14.6 ± 0.0^a^	3.0 ± 0.1^a^	19.5 ± 0.0^b^

∗All carotenoids except β-carotene were quantiﬁed using UHPLC-ESI-MS on the basis of their retention times compared with authentic standards; β-carotene was detected and quantiﬁed by UHPLC. DW, dry weight. Values represent mean ± SD of triplicate analysis (n = 3) with six replicates. Different letters (a-c) denote a significant difference between mean values within the same column.

Researchers have noted that red seaweeds generally lack a xanthophyll biosynthesis pathway after zeaxanthin, which results in an accumulation of zeaxanthin and lutein as major carotenoids [15] and agrees with the present finding. Consequently, this explains the minor presence of other carotenoids in this species. The β-carotene content of *E. denticulatum* (4.7 mg/100 g DW) was comparable to the previous findings of 5.4 mg/100 g DW of β-carotene detected in the red seaweed of the *Gracilaria spp* [37]. and 5.7 mg/100 g in *Kappaphycus alvarezzi* [38]. Interestingly, the tentative identification of other xanthophylls, such as rubixanthin, antheraxanthin, dinoxanthin, diatoxanthin and diadinoxanthin, in *E. denticulatum* (data not shown) adds a new perspective to the carotenoid profile of the red seaweeds of this species. In the present study, however, an exact quantification analysis of the abovementioned carotenoids based on pure compounds could not be carried out due to the absence or costliness of the pure standard. Further research is needed for the exact determination of these carotenoids. Authentication of this finding may contribute new information to the *E. denticulatum* carotenoid profile.

Fucoxanthin (2.76 g/100 g DW) is the predominant carotenoid found in the brown seaweed *S. polycystum*, as well as in most other brown seaweeds [15]. Consequently, the finding of a high fucoxanthin content in *S. polycystum* adds to the value and increases the potential commercial prospects of this seaweed, which currently remain underexploited. Moreover, this species is abundantly available along coastal Malaysia, and no firm commercial application has been established in Malaysia or elsewhere. In general, the fucoxanthin found in brown seaweed is in the form of trans-fucoxanthin. Abidov et al. [39] reported that a 2.4 mg of fucoxanthin intake per day can significantly increase the energy expenditure of obese female volunteers and promoted weight loss, decreased body fat, plasma triacylglycerol as well as liver lipid contents. Judging from the content of fucoxanthin in *S. polycystum* seaweed, the concentration will be sufficiently high to show the physiological activity of these substances when they are incorporated to general foods. The LC-MS/MS result correlates well with the HPTLC findings, which exhibited an intense, thick orange band (R_f_ = 0.39), denoting a high concentration. This finding proves the presence of fucoxanthin in *S. polycystum* seaweed, which suggests that it is responsible directly, partially or/and synergistically with other phenolic compounds for the anti-radical activity observed in the present study. Apart from fucoxanthin, appreciable amounts of astaxanthin, canthaxanthin, lutein and zeaxanthin were detected (at 25.5, 21.7, 11.7 and 13.9 mg/100 g DW, respectively). β-Cryptoxanthin was present in the lowest amount at 6.1 mg/100 g DW, while β-carotene was not detected in this species of seaweed. The carotenoid composition of *S. polycystum* obtained in the present study contradicts the finding of Motshakeri et al. [40] regarding the same seaweed. This previous study reported the presence of β-carotene (75 μg/100 g DW) but showed an absence of lutein, canthaxanthin and astaxanthin. In both studies, the carotenoids zeaxanthin, fucoxanthin and diatoxanthin were detected. Quantitation of carotenoids showed variation within the same species due to differences in sample preparation and possibly in the method and instruments used. However, this study [40] did not provide a clear explanation of the method used for quantitation. Hence, we could not make a comparison. Other compounds, such as diadinoxanthin, diatoxanthin and antheraxanthin, were tentatively identified in *S. polycystum* on the basis of their molecular mass (*m/z* 583, 567, and 585, respectively), as described in the literature [41, 42]. Since there were no reference compounds for these xanthophylls, the data could not be confirmed by authentic materials. Diatoxanthin is a xanthophyll commonly found in seaweed that contributes to the “diadinoxanthin cycle”. The presence of diadinoxanthin in seaweed is deemed a possible precursor for fucoxanthin synthesis [43]. Neoxanthin was not detected in *S. polycystum*, and this result is supported by Mikami and Hosokawa [15] due to the lack of a gene encoding neoxanthin synthase (NXS) in brown seaweed.

The highest carotenoid contents in *C. lentillifera* (green) were those of β-carotene and canthaxanthin (14.6 mg/100 g and 19.5 mg/100 g DW, respectively), while lutein and fucoxanthin were not detected, which is in accordance with the HPTLC finding. The other xanthophylls detected in this seaweed, zeaxanthin, β-cryptoxanthin and astaxanthin, had contents ranging between 1.3 and 3.0 mg/100 g, which agrees with the findings of Young et al. [44]. Zeaxanthin, found in *C. lentillifera*, is a common carotenoid present in other green seaweeds and higher plants. However, in contrast to terrestrial plants, green seaweed possesses extra xanthophylls, such as canthaxanthin and astaxanthin, as detected in *C. lentillifera*. Neoxanthin and violaxanthin were not detected in the tested green seaweed (*C. lentillifera*), as expected. The detection was based on comparing the mass and retention time with the available standards (cis-neoxanthin and all-trans-violaxanthin). Contrarily, other studies have reported the presence of violaxanthin and/or neoxanthin as prominent carotenoids in green seaweed [29, 45]. Nevertheless, it is worth noting that an unknown peak was detected at *m/z* 601 at a different retention time (RT = 19.14 min) from those of the violaxanthin (RT = 18.93 min) and neoxanthin (RT = 17.42 min) standard peaks. The detected compound was more nonpolar and eluted later than the violaxanthin peak from the referral standard. Further structural analysis is needed to verify this compound.

Table 3 shows the tentatively detected carotenoids in each species of seaweed, with some of the compounds having been confirmed with standards. Twelve carotenoid compounds were detected in the studied seaweeds, some for the first time. *E. denticulatum* displayed a wide variety of carotenoid contents compared to the other two species. *C. lentillifera* had the fewest detected pigments.

**Table 3:** Carotenoids detected in the studied seaweeds.

Pigment	Seaweed		
	*E. denticulatum*	*S. polycystum*	*C. lentillifera*
Antheraxanthin	+	+	-
Astaxanthin∗	+	+	+
β-Carotene∗	+	-	+
β-Cryptoxanthin∗	+	+	+
Canthaxanthin∗	-	+	+
Dinoxanthin	+	+	-
Diatoxanthin	+	+	-
Diadinoxanthin	+	+	-
Fucoxanthin∗	+	+	-
Lutein∗	+	+	-
Lycopene∗	-	-	-
Neoxanthin∗	-	-	-
Rubixanthin	+	-	-
Violaxanthin∗	-	-	-
Zeaxanthin∗	+	+	+

‘+’ denotes presence; ‘˗’ denotes not detected; asterisk denotes compound confirmed with standard.

From this analysis, it is evident that high-resolution mass spectrometry enables the determination of accurate masses with <5 ppm mass accuracy and detection of targeted compounds. An additional route to compound identiﬁcation is provided by the fragmentation of ions produced in a higher-energy collision dissociation (HCD) cell. Because of the limitations of the ESI source, it was not suitable for detecting the carotenes, and hence, UHPLC was used to quantitate β-carotene in this study.

### 3.3 Validation of parameters for carotenoid quantitation

The linearity of the developed methods was evaluated by preparing five-point calibration curves in solvents for the various carotenoids analyzed. The determination coefficients (R^2^) obtained for these compounds all exceeded 0.97, which suggests good linearity. The relative recoveries of astaxanthin, β-carotene, canthaxanthin, fucoxanthin, lutein, and zeaxanthin ranged from 85 % to 104 % (Table 4) at the level tested. However, the relative recovery of β-cryptoxanthin was the lowest at 74 %. This low recovery may be attributed to the β-cryptoxanthin instability, which was noted to be higher than those of other carotenoids, and to the lower solubility of this carotenoid in the injection solvent. Except for β-cryptoxanthin, satisfactory recoveries were obtained for the tested analytes. Nevertheless, according to Rivera et al. [46], the selection of the injection solvent was a compromise between the satisfactory solubility of carotenoids, the compatibility with the mobile phase, and the absence of peak distortions. Retention time repeatability was checked with 6 successive runs of the targeted analytes. The limit of detection (LOD) and lower limit of quantitation (LOQ) were determined based on the signal-to-noise ratio (S/N) of the analytes. The lowest LOD and LOQ values were 0.0011 and 0.0044 ng/μl, respectively, obtained for canthaxanthin, compared to 0.0418 and 0.1393 ng/μl, respectively, for β-carotene, for the UHPLC-DAD performance. The results obtained in this work attest to the performance of the high-resolution Orbitrap-MS with respect to ESI.

**Table 4:** Performance criteria for Orbitrap-MS.

Seaweed	LOD (ng/μl)	LOQ (ng/μl)	R^2^	Linear range (ng/μl)	Recovery (%)
Lutein	0.0170	0.0567	0.9966	0.0–1.0	99
Zeaxanthin	0.0017	0.0057	0.9731	0.0–1.0	99
Fucoxanthin	0.0014	0.0047	0.9995	0.0–2.0	104
β-Cryptoxanthin	0.0067	0.0150	0.9993	0.0–0.5	74
Canthaxanthin	0.0011	0.0044	0.9870	0.0–1.0	93
Astaxanthin	0.0023	0.0058	0.9975	0.0–1.0	88
∗β-Carotene	0.0418	0.1393	0.9981	0.0–1.0	85

∗Performance based on UHPLC.

### 3.4 Antioxidant capacity of *E. denticulatum*, *S. polycystum* and *C. lentillifera*

The inhibition of radical scavenging activity by the ethanolic extracts of *E. denticulatum*, *S. polycystum* and *C. lentillifera* (200 μg/ml respectively) is reported in Table 5. The results indicate that the ethanolic extract of *S. polycystum* (200 μg/ml) had the highest radical scavenging capacity (20.36 ± 0.7 %) among the three seaweeds. The scavenging capacities of *E. denticulatum* and *C. lentillifera* were considerably lower at 7.99 ± 0.5 % and 5.74 ± 0.9 %, respectively. This finding supports the previous reports on brown seaweed having better DPPH scavenging activity than red seaweeds [47, 48]. Furthermore, fucoxanthin, a nonphenolic carotenoid that is commonly found in brown seaweed, also exerts good DPPH radical scavenging activity [49].

**Table 5:** Antioxidant capacity of seaweed ethanolic extracts as determined by DPPH and ORAC.

Sample	DPPH (%)	ORAC (μmol TE/100 g)
*E. denticulatum*	7.99 ± 0.5^a^	112,762 ± 8,575^a^
*S. polycystum*	20.36 ± 0.7^b^	42,060 ± 5,153^b^
*C. lentillifera*	5.74 ± 0.9^a^	68,372 ± 1,596^c^

DPPH expressed as a percentage of radical scavenging activity, and ORAC expressed in μmol trolox equivalent per 100 g. Data are expressed as mean ± standard error mean (SEM) (n = 3). Different letters (a-c) denote a significant difference between mean values within the same column.

Conversely, the antioxidant capacity determined by ORAC assay (Table 5) showed that the *E. denticulatum* (112,762 ± 8,575 μmol TE/100 g) extract had the highest antioxidant ability against the peroxyl radicals with significant differences between species, while *S. polycystum* (42,060 ± 5,153 μmol TE/100 g) had the lowest, which explains the various other mechanisms of antioxidant. ORAC assays directly measure the scavenging capacity against the peroxyl radical, which is the exiting and emission of 2, 2′-azobis (2-methylpropionamidine)-dihydrochloride (AAPH). The ORAC values of *E. denticulatum* in the present study were comparable to those reported in the literature, including those of a 70% acetone extract of *Ascophyllum nodosum* (141,700 μmol TE/100 g extract) and *Laminaria hyperborea* (97,500 μmol TE/100 g extract) [50].

## 4 Conclusions

The HPTLC spectrum displayed a distinct carotenoid pattern for each class of seaweed, which reflects the potential for different effects on health. By using the LC–MS/MS technique, the molecular masses of carotenoids can be determined, and their structure can be explored based on the fragmentation pattern from collision-induced dissociation. Twelve types of carotenoids were detected in the seaweed ethanolic extracts based on the calculated mass (*m/z*). The seaweeds also showed potential antioxidant properties and a peroxyl radical scavenging effect, which is at least partly contributed by the carotenoid presence in the extracts. Previous research suggested several potential health benefits of the selected seaweeds, and the carotenoids may play a pivotal role in the health-promoting activities. In general, these seaweeds appear to be a promising, sustainable ingredient in future functional food product development.

## Declarations

### Author contribution statement

Vimala Balasubramaniam, Lee June Chelyn: Conceived and designed the experiments; Performed the experiments; Analyzed and interpreted the data; Contributed reagents, materials, analysis tools or data; Wrote the paper.

Vimala Subramaniam: Performed the experiments.

Mohd Fairulnizal Mohd Noh, Iain Brownlee, Amin Ismail: Contributed reagents, materials, analysis tools or data.

### Funding statement

This work was supported by 10.13039/501100013885Ministry of Health Malaysia (NMRR-12-500-12439; 12-011).

### Competing interest statement

The authors declare no conflict of interest.

### Additional information

No additional information is available for this paper.

## Appendix A Supplementary data

Supplementary 1.docx Supplementary 1.docx
