# Supplementary material for: Carotenoid composition and antioxidant potential of Eucheuma denticulatum, Sargassum polycystum and Caulerpa lentillifera
Source: Heliyon. 2020 Aug 12;6(8):e04654. doi: 10.1016/j.heliyon.2020.e04654 (PMC7426577; doi:10.1016/j.heliyon.2020.e04654)
Supplement: Supplementary 1.docx [file mmc1.docx]

Supplementary 1


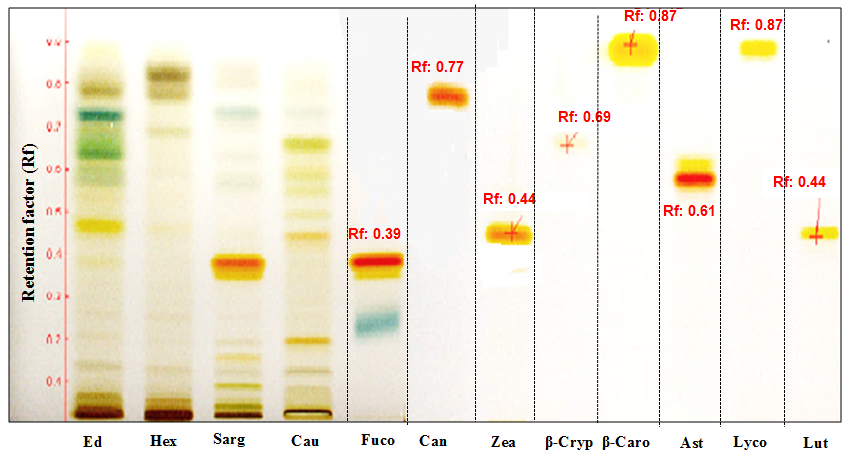


**Figure 1: High performance thin-layer chromatography (HPTLC) analyses of carotenoids from seaweed extracts and hexane fraction (Hex) and comparison with standards; Fuco (Fucoxanthin), Can (Canthaxanthin), Zea (Zeaxanthin); β-Cryp (β-Cryptoxanthin), β-Caro (β-Carotene), Ast (Astaxanthin), Lyco (Lycopene) and Lut (Lutein)**
